# Retraction: A Case of Accidental Autoerotic Death by Hanging Linked to Erotic Photography

**DOI:** 10.7759/cureus.r150

**Published:** 2024-10-30

**Authors:** Teodora Kiryakova, Biliana Mileva, Pavel Timonov, Antoaneta Fasova, Metodi Goshev, Alexandar Alexandrov, Ivan I Tsranchev

**Affiliations:** 1 Department of Forensic Medicine, Medical Institute of the Ministry of Internal Affairs, Sofia, BGR; 2 Department of Forensic Medicine and Deontology, Medical University, Sofia, Sofia, BGR; 3 Department of Forensic Medicine and Deontology, Medical University of Plovdiv, Plovdiv, BGR; 4 Department of Anatomy, Histology, and Embryology, Medical University of Plovdiv, Plovdiv, BGR

This article has been retracted by the Editor-in-Chief due to concerns over the nature of the consent obtained by the authors. Due to the sensitive nature of the article topic and concerns regarding potential identifiable information, the contents of the article have been removed. The authors agree with the decision to retract.

